# Hdh^Q111^ Mice Exhibit Tissue Specific Metabolite Profiles that Include Striatal Lipid Accumulation

**DOI:** 10.1371/journal.pone.0134465

**Published:** 2015-08-21

**Authors:** Jeffrey B. Carroll, Amy Deik, Elisa Fossale, Rory M. Weston, Jolene R. Guide, Jamshid Arjomand, Seung Kwak, Clary B. Clish, Marcy E. MacDonald

**Affiliations:** 1 Center for Human Genetic Research, Massachusetts General Hospital, Department of Neurology, Harvard Medical School, Boston, Massachusetts, United States of America; 2 Behavioral Neuroscience Program, Department of Psychology, Western Washington University, Bellingham, Washington, United States of America; 3 The Broad Institute of Harvard and MIT, Cambridge, Massachusetts, United States of America; 4 CHDI Foundation, Inc., Princeton, New Jersey, United States of America; Institut Curie, FRANCE

## Abstract

The *HTT* CAG expansion mutation causes Huntington’s Disease and is associated with a wide range of cellular consequences, including altered metabolism. The mutant allele is expressed widely, in all tissues, but the striatum and cortex are especially vulnerable to its effects. To more fully understand this tissue-specificity, early in the disease process, we asked whether the metabolic impact of the mutant CAG expanded allele in heterozygous B6.Hdh^Q111/+^ mice would be common across tissues, or whether tissues would have tissue-specific responses and whether such changes may be affected by diet. Specifically, we cross-sectionally examined steady state metabolite concentrations from a range of tissues (plasma, brown adipose tissue, cerebellum, striatum, liver, white adipose tissue), using an established liquid chromatography-mass spectrometry pipeline, from cohorts of 8 month old mutant and wild-type littermate mice that were fed one of two different high-fat diets. The differential response to diet highlighted a proportion of metabolites in all tissues, ranging from 3% (7/219) in the striatum to 12% (25/212) in white adipose tissue. By contrast, the mutant CAG-expanded allele primarily affected brain metabolites, with 14% (30/219) of metabolites significantly altered, compared to wild-type, in striatum and 11% (25/224) in the cerebellum. In general, diet and the CAG-expanded allele both elicited metabolite changes that were predominantly tissue-specific and non-overlapping, with evidence for mutation-by-diet interaction in peripheral tissues most affected by diet. Machine-learning approaches highlighted the accumulation of diverse lipid species as the most genotype-predictive metabolite changes in the striatum. Validation experiments in cell culture demonstrated that lipid accumulation was also a defining feature of mutant Hdh^Q111^ striatal progenitor cells. Thus, metabolite-level responses to the CAG expansion mutation *in vivo* were tissue specific and most evident in brain, where the striatum featured signature accumulation of a set of lipids including sphingomyelin, phosphatidylcholine, cholesterol ester and triglyceride species. Importantly, in the presence of the CAG mutation, metabolite changes were unmasked in peripheral tissues by an interaction with dietary fat, implying that the design of studies to discover metabolic changes in HD mutation carriers should include metabolic perturbations.

## Introduction

Huntington’s disease (HD) is a progressive autosomal dominant neurodegenerative disorder, caused by an expansion of a coding CAG tract near the 5’ end of *HTT* [1]. The disease is marked by a triad of cognitive, affective and diagnostic motor signs, and progresses inevitably to death, generally 10–15 years after onset [2]. Long term deep phenotyping of HD mutation carriers, both before and after onset of clinically diagnosable signs, reveals a suite of very specific behavioral and neuroanatomical changes that gradually increase in severity [3]. Cerebral structures, particularly the striatum and cortex, show the earliest and most evident atrophy, and dysfunction of cortico-striatal circuits is hypothesized to underlie many of symptoms in mutation carriers [3]. While HD is associated with a clear neuropathological signature, both wild type and mutant *HTT* alleles are widely expressed throughout the body, including peripheral organs [4]. Examination of HD mutation carriers also reveals a number of functional and structural alterations in peripheral tissues—notably evidence that prodromal mutation carriers tend to be thinner, despite increased caloric intake [5], an observation that has also been made in patients with frank symptoms of HD [6–8]. Population studies reveals that HD mutation carriers have a reduced risk of developing a wide-range of peripheral tumors [9], HD mutation carriers exhibit enhanced immune responses in peripheral monocytes and macrophages [10, 11] and progressively impaired hepatic mitochondrial function [12, 13].

Impaired energy metabolism is amongst the broad array of cellular aberrations proposed to contribute to HD pathogenesis [14]. Notably, ingestion of 3-nitropropionic acid, an irreversible inhibitor of mitochondrial succinate dehydrogenase/Complex-II, causes specific neuropathology reminiscent of HD [15, 16], leading to the hypothesis that the specific pattern of neuropathology in HD may be due to the unique sensitivity of these tissues to energetic compromise [17]. Direct molecular evidence suggests that mutant huntingtin reduces expression of the transcriptional co-activator PGC-1a [18, 19] whose activity is important for mitochondrial biogenesis, and increases mitochondrial fragmentation via potentiating interactions with DRP-1 [20–22]. A wide range of evidence reveals that the CAG expansion mutation is associated with reduced levels of ATP in human cells [23] and tissues from mouse models [24]. Positron emission tomography of the striatum of human mutation carriers reveals reduced glucose uptake [25–30] and impaired glycolysis predating disease onset [25, 31]. These phenotypes suggest that metabolic alterations may be an important part of the etiology of HD, though to date relatively few studies have systematically examined a range of peripheral tissues from either mouse models or human subjects, in addition to brain tissues.

As part of an effort to map metabolically relevant phenotypes in heterozygous B6.*Hdh* ^*Q111/+*^ mice, which carry an expanded CAG repeat inserted at the proper location in the mouse *HTT* homolog (previously *Hdh*, now renamed *Htt*), we investigated whether stable metabolite concentrations were altered in the presence of the expanded CAG mutation across a range of tissues. Existing metabolic analyses of *Hdh* ^*Q111/+*^ mice fed regular chow reveals that they do not show body weight changes, compared to *Hdh* ^*+/+*^ mice [32], unlike full-length transgenic strains of mice expressing mutant HTT [33]. Deeper metabolic analysis of *Hdh* ^*Q111/+*^ mice reveals no changes in a range of important clinical chemistry parameters, including serum glucose, lipids, albumin, creatinine, ferritin, iron and lactate in mice fed a normal chow diet [34]. Given the effects of the mutation were expected to be subtle, we reasoned that observing metabolite changes may require physiological perturbation of *Hdh* ^*Q111/+*^ mice. To that end we utilized a metabolic perturbagen paradigm whereby mice were fed one of two different diets commonly utilized in obesity research reasoning that differential responses to related but distinct chronic metabolic stresses may be revealing of the early effects of the mutation. Our goal was to establish whether, at the metabolite level, there is a core set of “CAG-responsive” metabolites, or whether each tissue has a unique response to the mutation, and if this might be influenced by dietary fat.

## Results and Discussion

### Metabolite profiling reveals changes in all tissues tested, particularly the striatum

To quantify genotype-sensitive changes in stable metabolite concentrations in a systematic and unbiased way, we measured of steady-state levels of ~250 metabolites using 3 parallel liquid-chromatography tandem mass spectrometry (LC-MS) methods, which have been successfully applied to studies of human disease [35–38], in tissues from otherwise isogenic B6.*Hdh* ^*Q111/+*^ (n = 16) and wild-type littermate B6.*Htt* ^*+/+*^ mice (n = 14). Note that to provide continuity with the published literature, we here use *Hdh* ^*Q111*^ to refer to the name of the knock-in mouse line and *Htt* to refer to the locus. The size of the *Htt* CAG repeat knock-in allele in each *Hdh* ^*Q111/+*^ mouse was determined by specific PCR amplification assay as previously reported [39]. A summary of the cohort characteristics including diet, genotype and CAG size is provided in S4 File.

To analyze metabolites across tissues, we prepared lysates from 5 solid tissues (striatum, cerebellum, interscapular brown adipose tissue, perigonadal white adipose tissue and liver, normalized by wet weight) as well as plasma from mice were sacrificed at 236 +/- 7 days of age. Striatum and cerebellum were chosen as representative brain regions which are, respectively, relatively sensitive and resistant to the pathological effects of mutant *Htt* expression [40]. The study was designed as an endpoint study, by 7 months of age *Hdh* ^*Q111/+*^ mice display important features of disease, including memory deficits [41] as well as perturbed transcriptional and protein signatures compatible with disease (Carroll and MacDonald, unpublished observations). This endpoint design precludes an analysis of progressive changes, which have been observed in a more limited number of metabolites using magnetic resonance spectroscopy in this mouse model [42]. One half of the cohort were fed *ad libitum* from weaning with ‘high fat’ chow, with 60% calories from fat, 20% from carbohydrate and 20% from protein, whereas the other half of the cohort received isocaloric ‘medium fat’ chow with fewer calories from fat (45%), more from carbohydrate (35%) and equivalent calories from protein (20%). This analysis yielded a large dataset of 46,032 integrated LC-MS metabolite peak areas, with values for 169–255 metabolites of known identity for each sample (raw results provided in S1 File).

**Fig 1:**
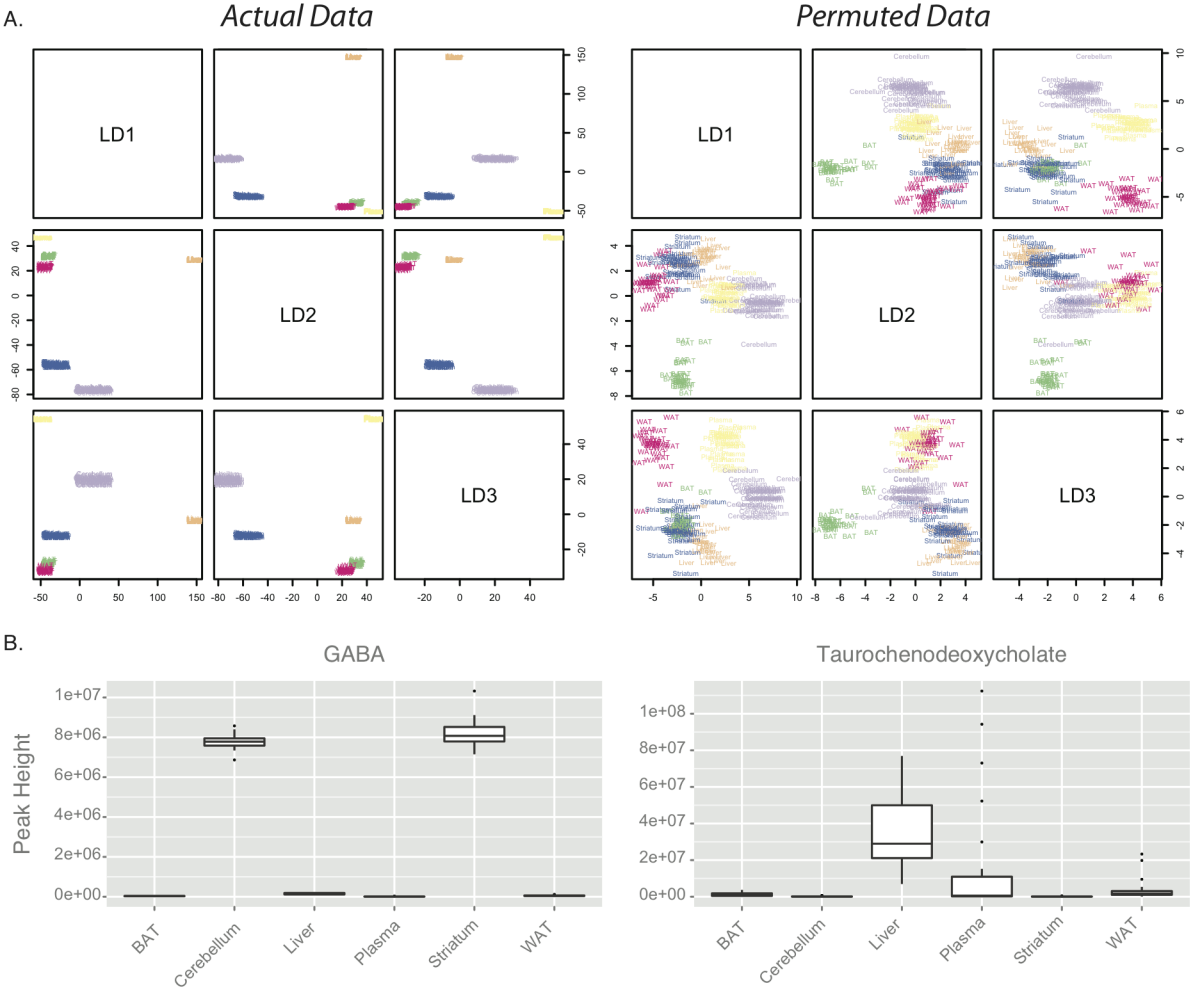
Metabolite profiles clearly discriminate tissue types. A—Linear discriminant analysis was conducted to derive linear combinations of metabolite concentrations that separate tissue types. In this approach, linear combinations of factors (here, metabolites) are constructed that maximally differentiate a factor of interest (here, tissue type). Scatter-plot matrix of the top 3 linear discriminants (linear combinations of variables) constructed reveals that they very effectively separate tissue types; the tissue type of each sample is indicated by text and color. To test the robustness of this finding, the tissue labels of each sample were randomly permuted, and the LDA analysis repeated. Increased scatter suggests that tissue-separation is much less accurate using permuted data, consistent with the hypothesis that inter-tissue variability is lower than intra-tissue variability in these samples. Linear discriminants derived from actual data are shown on the left, those derived from tissue-label permuted data are shown on the right. B—Sample concentrations of metabolites with clear tissue-specific roles; GABA is high in CNS tissues (striatum and cerebellum), and absent in peripheral tissues. The conjugated bile acid taurochenodeoxycholate are present in the liver, and to a smaller extent the plasma, but absent from other tissues.

Because a comprehensive profile of tissue polar metabolites and lipids from C57BL/6J mice has not previously been reported, we first examined the variation in metabolite concentrations across different tissues to determine whether, as expected, the inter-tissue variation would be larger than the intra-tissue variation, irrespective of genotype or diet. The results of linear discriminant analysis showed that pairs of the top 3 linear discriminants clearly separated each of the tissue types from one another, whereas when the tissue labels for all metabolite peak areas were permuted the results of the linear discriminant analysis did not as efficiently separate the tissue types, confirming the biological coherence of the dataset (Fig 1A). Other approaches, including hierarchical clustering, also demonstrated clear separation of tissue types by metabolite profiles (data not shown). The tissue specificity of the metabolite profiles for each tissue was further validated by manual inspection of metabolites known to vary across tissue types, based on biological function. For example, as expected, the neurotransmitter gamma-amino butyric acid (GABA) was highly enriched in the brain tissues (striatum and cerebellum), while high levels of the conjugated bile acids taurochenodeoxycholate or taurodeoxycholate were found only in the liver and plasma (Fig 1B). A confound induced by the sacrifice method (CO_2_ inhalation followed by rapid decapitation), is that labile metabolites may have experienced significant post-mortem changes [43]. This could be a particularly important confound for high-energy metabolites involved in central metabolism, while brief post-mortem intervals may be less of a confound for more chemically stable metabolites such as lipids.

**Fig 2:**
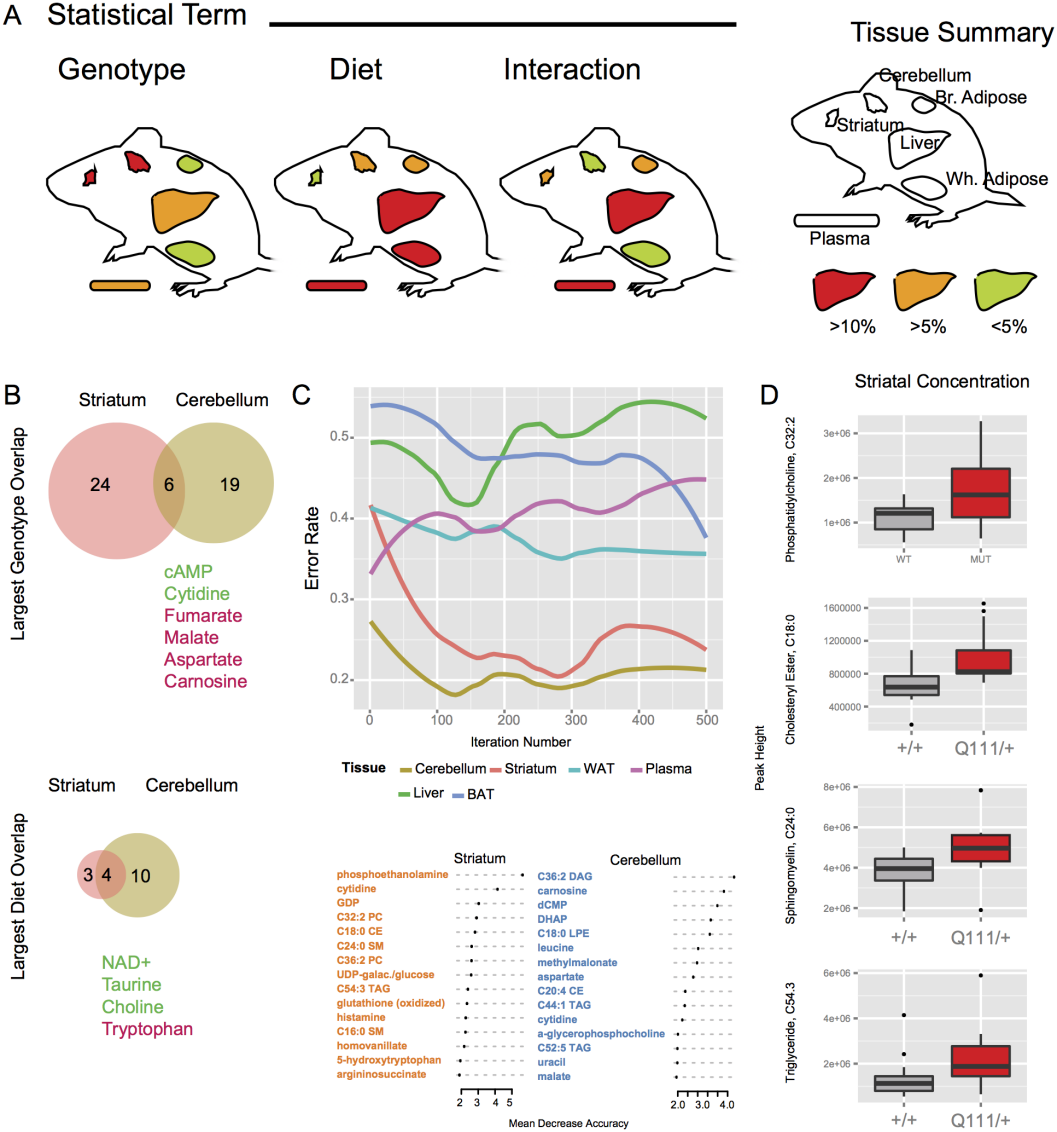
Alterations in tissue metabolite levels in response to CAG-expansion in *Htt* and increased dietary fat. A) Summary of the number of nominally significant metabolites per tissue (as a percentage of total measured metabolites) for each tissue—heat map color indicates the number of metabolites significant at p < 0.05. B) A relatively limited number of metabolites show concordant (green text) or discordant (red text) concentration changes in response to CAG-expansion in *Htt* in the striatum and cerebellum. The striatum and cerebellum also show the largest number (4) of overlapping diet-responsive metabolites amongst tissue pairs. C) Determination of genotype using metabolite concentrations using random forests—the error rate for each tissue is shown over 500 iterations. Below, the contribution of individual metabolites to model accuracy is shown for striatum and cerebellum, the two tissues with accurate models. The y-axis indicates the increase in errors in genotype prediction (in %) after permuting the genotype labels for the indicated metabolite. D) The concentrations of lipid species important for genotype prediction in the striatum are increased in the *Htt* ^*Q111/Q7*^ samples (PC 32:2–49% increase, p = 0.024; CE 18:0–54% increase, p = 0.004; SM 24:0–33% increase, p = 0.02; 65% increase, p = 0.06).

The genotype/two-diet study design that we utilized provided three comparisons of interest for each metabolite in each tissue: the effect of genotype (*Htt* ^Q111/+^ vs. *Htt* ^*+/+*^), the effect of diet (45% vs. 60% fat) and genotype-dietary fat interactions. To understand the effects of these 3 factors we constructed linear models relating the levels of each metabolite in each tissue to genotype, diet and an interaction term after filtering out metabolites with less than 3 non-0 values per arm of the study. The number of *Htt* genotype-sensitive metabolites nominally significant at an alpha level of 0.05 varied across tissues (Chi sq. (5) = 25.9, p<0.0001), ranging from 3% of measured metabolites in the white and brown adipose tissue to 14% in the striatum (Table 1, Fig 2A). While *Htt* genotype was associated with a greater number of metabolite alterations in the brain (both striatum and cerebellum) than in the periphery, increasing dietary fat had a larger impact on metabolites in the peripheral organs (Chi sq. (5) = 23.4, p<0.0001), ranging from 3% in the striatum to 21% in the plasma (Table 1, Fig 2A). The number of diet-*Htt* genotype interaction sensitive metabolites also varied across tissues (Chi sq. (5) = 23.4, p<0.0001), in a pattern closely mirroring trends observed for diet-induced changes, ranging from 3% in the cerebellum to 12% in the liver (Table 1, Fig 2A). These data are consistent with the view that chronic exposure to the CAG repeat expansion has some impact in most tissues and that effects of the mutation on peripheral metabolite concentrations in Hdh^Q111/+^ mice were uncovered by looking for metabolites that were sensitive to interactions between diet stressors and the *Htt* genotype.

**Table 1:** The number of measured metabolites sensitive to *Htt* genotype, diet or to an *Htt* genotype-diet interaction varies across tissues. For each tissue, metabolite values were filtered to include only those with at least 3 non-0 values per arm of the diet/genotype study design.

**Tissue**	**Genotype**	**Diet**	**Interaction**
**Striatum**	14% (30/219)	3% (7/219)	5% (11/219)
**Cerebellum**	11% (25/224)	6% (14/224)	3% (7/224)
**Liver**	5% (13/255)	11% (27/255)	12% (30/255)
**Plasma**	8% (19/169)	21% (36/169)	14% (23/169)
**White Adipose**	3% (7/212)	12% (25/212)	2% (4/212)
**Brown Adipose**	3% (7/228)	5% (11/228)	6% (13/228)
Ch. Sq., df	25.9, 5	23.4, 5	23.4, 5
p-value	< 0.0001	0.0003	0.0003

We were initially interested in metabolite changes that were common to all, or many of the tissues, reasoning that their existence may reveal fundamental tissue-independent responses to the CAG expansion mutation. The number of overlapping genotype- and diet-sensitive metabolites was limited (Fig 2B, Table 2). The largest group of similarly altered metabolites is *Htt* genotype-sensitive metabolites in the striatum (30 genotype-sensitive metabolites) and cerebellum (25 genotype-sensitive metabolites), which shared 6 coherent metabolite concentration changes (Fig 2B). Diet alone elicited metabolites altered in both the striatum (7 diet-sensitive metabolites) and cerebellum (14 diet-sensitive metabolites), which shared 3 coherent and 1 discordant metabolite concentration changes (Fig 2B).

**Table 2:** Counts of overlapping significant metabolites between tissue pairs, by factor.

**Genotype**						
	Striatum	Cerebellum	Liver	WAT	BAT	Plasma
Striatum						
Cerebellum	6					
Liver	2	1				
WAT	1	1	0			
BAT	0	1	1	2		
Plasma	1	0	0	0	0	
**Diet**						
	Striatum	Cerebellum	Liver	WAT	BAT	Plasma
Striatum						
Cerebellum	4					
Liver	3	2				
WAT	1	3	1			
BAT	0	1	0	2		
Plasma	0	0	2	0	2	
**Diet/genotype Interaction**						
	Striatum	Cerebellum	Liver	WAT	BAT	Plasma
Striatum						
Cerebellum	0					
Liver	1	2				
WAT	0	0	0			
BAT	0	2	1	0		
Plasma	0	1	0	1	2	

### A lipid-enriched set of genotype-predictive metabolites in the striatum

Given the special vulnerability of the striatum in HD and the apparent selective sensitivity of striatal metabolite concentrations to *Htt* genotype compared to the peripheral tissues (Table 1), we then focused on metabolites in striatum that were most sensitive to the presence of the expanded CAG *Htt* allele. A list of the top 10 genotype-sensitive metabolites in the striatum, ranked by the F-statistic derived from an analysis of variance (ANOVA) genotype term, is presented in Table 3. Amongst these metabolites there did not appear to be general patterns in either the direction of change or whether the *Htt* genotype-sensitive metabolite was enriched in the striatum compared to other analyzed tissues. For example, the concentration of some genotype-sensitive metabolites was higher in *Hdh* ^Q111/+^ striatum than *Hdh* ^+/+^ striatum (i.e. cytidine); others were lower (i.e. phosphoethanolamine). Some metabolites were sensitive to genotype only in the striatum, despite being of higher concentration in other organs (i.e. taurine in the liver), whereas other metabolites were uniquely enriched in the striatum and only sensitive to *Htt* genotype in this tissue (i.e. phosphoethanolamine).

**Table 3:** Striatal enrichment of the 10 most genotype-sensitive metabolites in the striatum of *Htt* ^*Q111/+*^ mice. Top ranked metabolites by genotype ANOVA F-statistic are included in order of magnitude. The relative integrated peak height of each metabolite in the striatum compared to the liver and cerebellum is indicated by the rank of each metabolite on lists of 243 total metabolites.

Metabolite (KEGG compound ID)	Rank, [Striatum]/[Liver] (of 244 metabolites)	Rank, [Striatum]/[Cerebellum (of 243 metabolites)	*Htt* ^*Q111/+*^ vs. *Htt* ^*+/+*^ [Striatum] direction of change
**Phosphoethanolamine (C00346)**	11	8	Decreased
**Cytidine(C00475)**	15	10	Increased
**Taurine (C00245)**	76	42	Decreased
**NAD+ (C00003)**	36	16	Increased
**Triiodothyronine (C02465)**	75	41	Decreased
**Cholesteryl Ester (C18:0) (C02530)**	147	33	Increased
**UDP-Galactose/UDP-Glucose (C00029/C0005)**	114	104	Increased
**L-2- Hydroxyglutaric acid (C03196)**	29	56	Decreased
**Carnosine (C00386)**	1	39	Decreased
**Asparate (C00049)**	9	188	Decreased

Given the apparent complexity of the response to the mutation and the difficulty of empirically ranking the importance of specific metabolic changes, we utilized the ‘random forest’ algorithm (28) to identify those metabolites from amongst all metabolites measured in a particular tissue that contributed maximally to predicting the true *Htt* genotype (heterozygous mutant or wild-type). Briefly, this technique samples a sub-set of a large number of measured factors (metabolites, in this case) and constructs a classification tree to predict a factor of interest (genotype). Over a series of 500 iterations, the success rate of each classification tree is compared to the previous best tree, resulting in a consensus based classification tree. This consensus tree is cross-validated using unsampled data, providing an objective measure of its accuracy. The metabolite data for each tissue were considered separately, and Fig 2C summarizes the accuracy of the random forest derived tree over 500 iterations. In general, the classification tree accuracy in assigning genotype from the metabolite profiles was near chance (error rate of 0.5) when the data from each of the peripheral organs was used. However, accurate classifications were achieved using the brain tissue metabolite values. In the striatum and cerebellum, the brain regions examined, the classification tree approached 80% accuracy when predicting genotype from metabolite measurements (Fig 2C). The random forest approach also enables ranking the importance of individual factors (metabolite concentration values) to discerning the factor of interest (genotype) in an objective manner. For each tissue’s large set of metabolite concentrations, genotype levels are permuted for each metabolite in turn, and the random forest reconstructed with the permuted data. Comparing the original accuracy rate to the genotype-permuted accuracy rate allows metabolite ranking in an objective, model-independent manner. The subsets of metabolites contributing to the classification trees for striatum and cerebellum are shown in Fig 2C, as only the metabolite datasets for these two brain tissues discriminated sample genotype with a high level of accuracy.

The top-ranked metabolite for discriminating *Hdh* ^Q111/+^ from wild-type striatum was phosphoethanolamine (18% reduced in the former), an important intermediate in *de novo* phosphatidylethanolamine (PE) synthesis. Consistent with our observations, levels of phosphoethanolamine are reduced in the caudate of HD patients and the temporal lobes of Alzheimer’s post-mortem tissue samples [44]. Recently, the anti-histamine drug meclizine has been shown to shift cells from oxidative to glycolytic energy production [37], and also to protect mouse striatal progenitor cells, and model organisms, from polyglutamine-fragment induced toxicity [45]. The proposed mechanism of action driving meclinize-treated cells towards glycolytic metabolism is inhibition of the cytoplasmic enzyme CTP:phosphoethanolamine cytidylyltransferase (PCYT2), leading to an accumulation of its respiration-inhibiting substrate, phosphoethanolamine [46]. If this model is correct, our results would predict reduced inhibition of respiration by phosphoethanolamine, and a relative shift from glycolytic to oxidative metabolism in the striatum of heterozygote *Hdh* ^*Q111*^ mice.

Amongst the remaining genotype-predictive metabolites in the striatum were a number of lipid species. Indeed, 6/10 (60%) of the most genotype-discriminatory metabolites in this tissue were lipid species (Fig 2C). The concentration of each lipid species highly ranked by the random forest was increased in the striatum of *Hdh* ^Q111/+^ mice, compared to wild-type mouse striatum (Fig 2D). These lipids included triglyceride, sphingomyelin, phosphatidylcholine and cholesteryl ester species with variable chain lengths. Individual species chosen by the algorithm are indicated in Fig 2, but the trends represented by these individual species appear to be more general. The concentrations of these same species in the cerebellum were either unchanged or were slightly decreased, suggesting that genotype-specific lipid accumulation may be a unique feature of the Hdh^Q111/+^ striatum at this age.

Another study using overexpression of full-length mutant *Htt* in primary striatal neurons observed an increase in total cholesterol content, consistent with our findings of increased cholesterol esters in the striatum, along with other lipid species [47]. Gene expression studies have noted a coordinate down-regulation of lipid biosynthetic genes in cells expressing CAG expanded *Htt* and in cells that lack *Htt* expression [48]. Interestingly, pathway analysis [49] with gene sets derived from curated biological pathways (including pathways from Biocarta, KEGG, BioCyc and Gene Ontology) of transcriptomic data suggests that CAG expansion and *Htt* null mutations both alter processes involved in cholesterol/lipid metabolism, despite the fact that at the single gene level there is no significant overlap in the effects of the distinct *Htt* mutational paradigms [50]. These results suggest that CAG expansion in *Htt* causes alterations to specific lipid metabolic pathways that normally require *Htt* function.

Reduced lipid biosynthetic transcripts in HD may result from transcriptional dysregulation, or alternatively, lipid accumulation induced by some effect of expression of mutant *HTT* may lead to an appropriate homeostatic down-regulation of lipid biosynthetic genes. In support of the latter hypothesis, increased accumulation of neutral lipids in lipid droplets, stained with Oil red O, occurs in the striatum and fibroblasts of human HD patients, a phenotype that has been attributed to defective selective macroautophagy [51]. Impaired macroautophagy has been hypothesized to contribute to HD pathogenesis [52], and inhibition of autophagy increases lipid storage while reducing beta-oxidation of lipids [53, 54]. Interestingly, in light of the coordinated regulation of autophagy and lipid catabolism, cells from mice engineered to lack any polyglutamine-encoding codons in exon 1 of the endogenous *Htt* locus have both increased ATP levels and increased signs of autophagy, supporting a model in which the glutamine tract in *HTT* acts to negatively regulate both energy production and autophagy [55]. In this attractive scenario, the accumulation of cholesterol esters and triacylglycerol species in the *Hdh* ^*Q111*^ striatum in our metabolite profiling study could reflect reduced autophagic flux and concomitant increases in neutral lipid storage. Alternatively, the observation that various lipid species are affected in the *Hdh* ^*Q111*^ striatum could also reflect important changes in the regulation of common lipid synthetic pathways.

### STHdh^Q111^ striatal progenitor cells exhibit lipid accumulation

**Fig 3:**
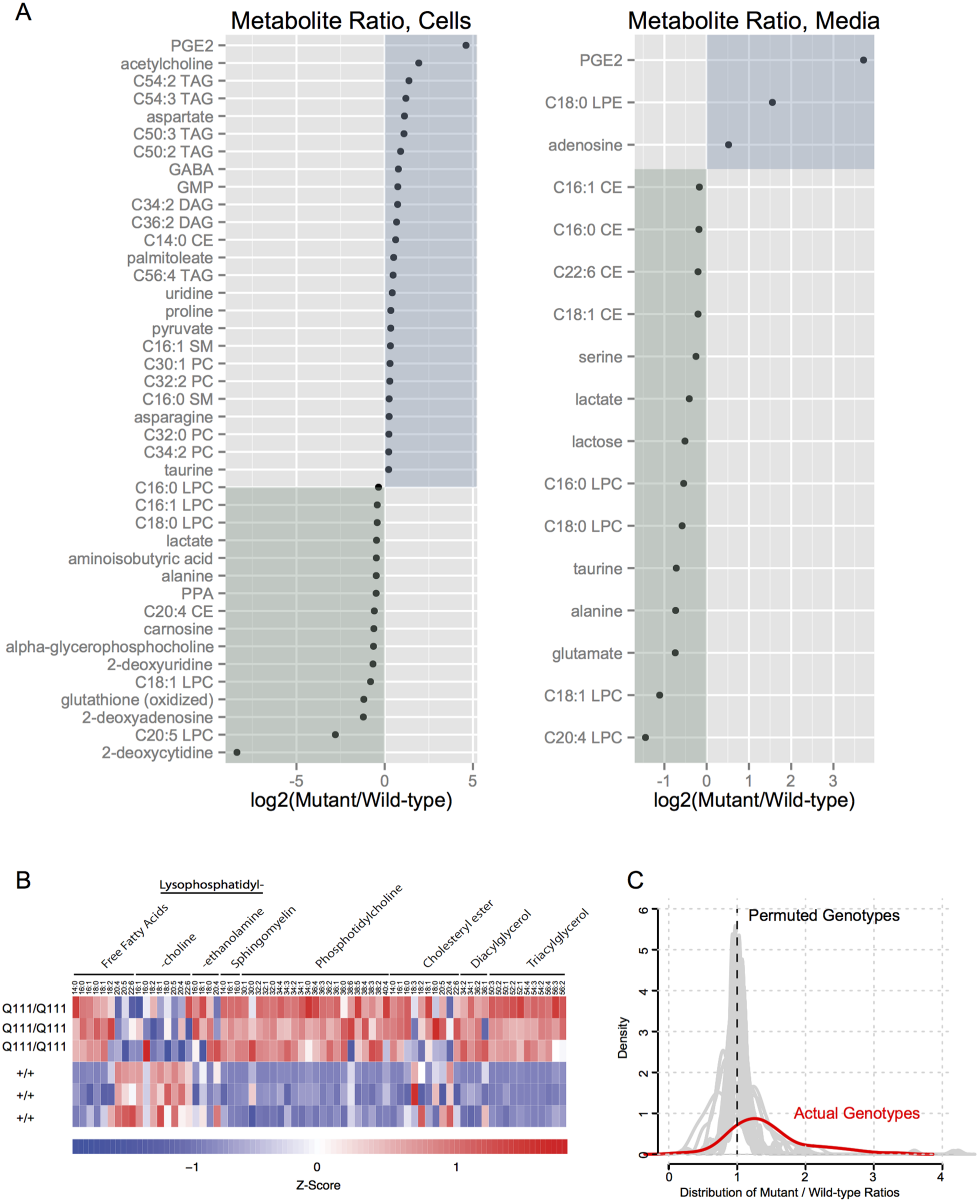
Increased lipid concentrations are common in *STHdh* ^Q111/Q111^ cell pellets. A) Strip-plots summarize the logarithm of the concentration ratios of metabolites altered in mutant, compared to wild-type, in cell pellets (left) or growth media (right) at a false-discovery rate of 10% and fold-change cut-off of +/- 30%. Metabolites whose concentrations are increased in mutant samples are indicated with a blue square, while those metabolites that are decreased in mutant samples are shown in a green. B) Normalized lipid peak values in replicate *STHdh* ^+/+^ and *STHdh* ^Q111/Q111^ cell pellets are depicted in the heat-map with higher concentrations in red and lower concentrations in blue. For comparison across lipid species, LC-MS peak heights were converted to a Z-score by subtracting the mean concentration of each species and dividing the remainder by the standard deviation. Each lipid class includes species with variable chain lengths and number of saturated bonds (columns). With the exception of lysophosphatidylcholine species, most lipid species are increased in mutant cells. C) The distribution of the actual mutant / wild type peak ratios for all measured lipid concentrations is shown in red. To test for lipid accumulation in mutant cells, the same distribution was calculated 1,000 using the same data with permuted genotype labels; those distributions are shown in gray.

To assess the possibility that the same *Htt* CAG expanded allele harbored by *Hdh* ^*Q111*^ mice may exert cell-autonomous effects, we also performed metabolite profiling of cultured immortalized homozygote ST*Hdh* ^*Q111/Q111*^ striatal cells, and their wild-type littermate ST*Hdh* ^*+/+*^ counterparts [56], as well as their respective growth medium. Note that *in vitro* we are using homozygous ST*Hdh* ^*Q111/Q111*^ striatal cells, while our *in vivo* data was generated with heterozygous *Hdh* ^*Q111/+*^ tissues. Despite this limitation, we believe this to a useful comparison, because previous work has shown that heterozygous ST*Hdh* ^*Q111/Q7*^ striatal cells exhibit dominant energetic deficits [23, 57, 58], compared to the wild-type striatal cells. Three independent cell cultures for each genotype were grown to mid-log phase in standard glucose-, pyruvate- and glutamine-containing DMEM with fetal calf serum and the concentrations of a large number of metabolites were measured using LC-MS/MS, in both the cell pellets and the corresponding growth media. The metabolite values obtained from these measurements are summarized in S2 File, with metabolite metadata presented in S3 File. The subset of genotype-sensitive metabolites was determined by performing ANOVA after fitting a linear model considering genotype as a factor for each metabolite. The logarithm of the concentration ratio for each metabolite in cells (ST*Hdh* ^*Q111/Q111*^ over ST*Hdh* ^*+/+*^) is summarized in Fig 3A; depicted are those metabolites significant at false discovery rate of 10%, with at least a 30% fold-change. About 20% of the cell metabolites measured (41 of 185 metabolites, Fig 3A) were genotype-sensitive, whereas only 9% (17 of 185 metabolites, Fig 3A) measured in the spent growth media for each cell replicate were different by genotype.

Alterations in a large number of lipid species were observed, particularly in cell pellets (Fig 3A). To examine this more closely, we normalized the peak values of each lipid species, the Z-scores for each lipid species quantified *in vitro* are depicted in Fig 3B. We next considered the entire set of lipid concentration genotype ratios from cell pellets (for each lipid species, considering the ST*Hdh* ^*Q111/Q111*^ / ST*Hdh* ^*+/+*^ value). The distribution of these values is graphed in red in Fig 3C, which reveals that these values cluster at a value greater than 1, consistent with lipid accumulation. To confirm this, we next permuted genotype labels from our lipid concentration ratios 1,000 times and graphed the resulting distributions in gray (Fig 3C). The distribution of these permuted datasets has a mean around 1, consistent with no accumulation in randomized data.

Of examined lipid species in ST*Hdh* ^*Q111/Q111*^ cells, most were increased compared to ST*Hdh* ^*Q7/Q7*^ cells. Notable exceptions are the concentrations of lysophosphatidylcholine species, which are generally decreased in ST*Hdh* ^*Q111/Q111*^ cells compared to ST*Hdh* ^*Q7/Q7*^ cells. This decrease, coupled with the overall increase in phosphatidylcholine species, is consistent with reduced activity of phospholipase-A2 (PLA2) enzymes, which cleave phosphatidylcholine species at the sn-2 position, yielding free fatty acids and lysophosphatidylcholine. Polyunsaturated fatty acids are preferentially found at the sn-2 position, and freed by PLA2 activity with diverse functional consequences. In ST*Hdh* ^*Q111/Q111*^ cells, polyunsaturated free fatty acids (including eicosatetraenoic acid 20:4, eicosapentaenoic acid 20:5 and docosahexaenoic acid 22:6) are decreased, while shorter chain (C14-18) free fatty acids accumulate in (Fig 3B). Free palmitatic acid (16:0), for example, is 30% increased in ST*Hdh* ^*Q111/Q111*^ cells, while free arachidonic acid (C22.6) is 13% decreased, compared to ST*Hdh* ^*Q7/Q7*^ cells. These *in vitro* findings suggest potential alterations in phospholipid signaling in ST*Hdh* ^*Q111/Q111*^ cells.

**Fig 4:**
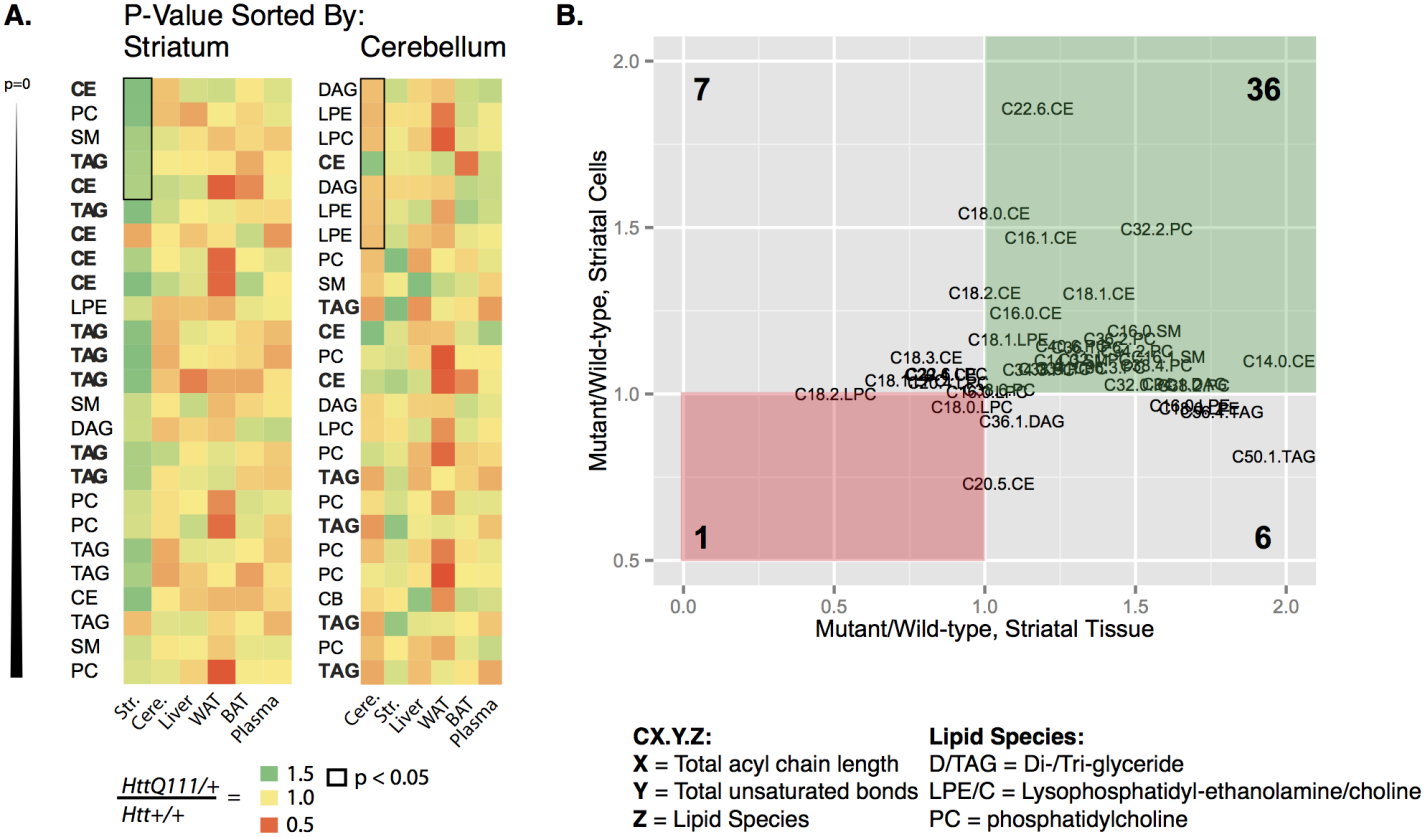
Coherently increased lipids in striatal cells and striatal tissue from *Hdh* ^*Q111/+*^ mice. A. The mutant/wild-type ratio of each lipid species is indicated on the heatmap (red = 0.5, yellow = 1.0 and green = 1.5). In the panel on the left, lipid species (rows, labels omitted for clarity) were sorted by their genotype p-value in the striatum. Sorting in this way reveals a clustering of green boxes near the top of the heatmap, suggesting an enrichment of accumulated lipids in the striatum. In the panel on the right, the same data was sorted by the genotype p-value in the cerebellum—no such clustering is observed. Metabolites nominally significant in a given tissue are outlined in black. The class of each lipid is indicated in the text, with neutral lipid species indicated in bold. B. The mutant/wild-type ratio of each lipid species that was quantified in both striatal tissue and striatal cells is graphed for striatal tissue (horizontal axis) and immortalized striatal cells (vertical axis); values greater than 1 indicate increased concentrations in mutant samples, while values less than 1 indicate decreased concentrations in mutant samples. The ratios in both cells and tissue for each lipid species are indicated by the position of its label. Lipids whose levels are increased in both striatal cells and tissue predominate, and are indicated by the green rectangle. Lipids with inconsistent changes between cells and tissue are in gray rectangles, while only 1 measured lipid species (C18.0.LPC) is decreased in both samples. Lipid species are indicated as CX.Y.Z, where X = total acyl chain length, Y = total number of unsaturated bonds and Z = lipid species. The lipid species graphed are D/TAG = Di-/Tri-glyceride; LPE/C = Lysophosphatidyl-ethanolamine/choline; PC = phosphatidylcholine.

In light of many increased lipid concentrations *in vitro* and *in vivo*, we next considered whether lipid accumulation was a general feature of tissues from *Hdh* ^*Q111/+*^ mice, or whether this phenotype was specific to the striatum. The heat map in Fig 4A describes the relative concentration of each lipid species in each tissue by displaying the mutant / wild-type ratio (color code: red = 0.5, yellow = 1, green = 1.5). When all the lipid species in each tissue are ordered by their striatal genotype effect p-value (Fig 4A, left panel, only the top 33 lipids shown), a clear clustering of green is noted near the end of the heat map corresponding to the lowest p-values. When the same dataset is sorted by the genotype effect p-value in the cerebellum (Fig 4A, right panel), no such clustering is observed, suggesting that the *coherence* of lipid accumulation is a novel feature of the Hdh^Q111/+^ striatum. Those lipid species whose concentration is nominally significant at p < 0.05 are outlined in black for each tissue. Finally, in order to compare our *in vitro* and *in vivo* findings, we graphed the mutant/wild-type concentration ratio for each lipid species whose levels were quantified in both the cultured ST*Hdh* ^*Q111*^ striatal cells and in the *Hdh* ^*Q111*^ striatal tissue (50 species, Fig 4B). Comparison of the distribution of our data with an evenly distributed number of lipid species per quadrant supports the hypothesis that a large number of lipid species are increased in both striatal cells and striatal tissues with this mutant CAG expansion allele (actual quadrant distribution vs. theoretical even distribution: chi-squared(3) = 24.6, p < 0.0001).

These data are consistent with the hypothesis that the CAG expanded *Htt* allele in striatal cells is associated with altered lipid metabolism, or changes in membrane structure that reveal themselves as altered lipid concentrations. One concern with striatal-specific tissue-level data from *Hdh* ^Q111/+^ mice at 8 months of age is the potential confound of altered tissue composition due to neuritic retraction and/or cell shrinkage. The good agreement between striatal tissue and striatal cells that harbor the same knock-in CAG mutation (Fig 4) suggests that lipid accumulation is a cell autonomous phenotype, and doesn’t appear to originate in altered striatal tissue composition in *Hdh* ^*Q111/+*^ mice.

## Conclusions

Knowledge of the dominant effects of the *HTT* CAG repeat can refine the understanding of the disease process triggered by the mutation that is the root cause of all cases of HD. Observations of altered measures of metabolism in carriers of expanded *HTT* CAG alleles, as well as in individuals with these alleles who exhibit overt clinical symptoms, strongly support the hypothesis that *HTT* is a negative regulator of cellular energy levels in a manner that is modulated by the size of the CAG tract. These energetic changes may contribute early in the disease process, either as a causative factor or as a compensatory response. The results of our systematic analysis of several hundred metabolites across a panel of tissues clearly demonstrated that single *Htt* CAG expansion allele, expressed at endogenous levels, is sufficient to induce significant changes in the concentration of metabolites in a number of tissues in adult mice after chronic metabolic stress. These changes are largely tissue-specific and are most pronounced in the striatum, as judged by the number of altered metabolites, but are observed to varying degrees in a number of non-brain tissues. Interestingly, again judged by numbers of metabolites affected, the response of the striatum to the CAG repeat allele was about on par with the response of liver tissue and plasma to the different high fat diet stressors. In the mutant striatum and mutant striatal cells, the accumulation of diverse lipid species provides a novel striatal-specific metabolite signature. A limitation of our study is that we did not perform metabolic profiling in HdhQ111 mice under a baseline standard diet that would have allowed comparing the metabolic changes induced by the diet itself. However, our study revealed differential responses to related high fat-diet stressors, which encourages study designs that include a perturbation arm. Indeed, a number of the metabolite changes elicited by the CAG expansion mutation in knock-in mice are amenable to follow up study in HD patients and may serve as readily detectible markers of various aspects of the early pathogenic process.

## Materials and Methods

### Cell Culture

The creation of temperature-sensitive immortalized striatal progenitor cells from STHdh^Q111/111^, STHdh^Q111/+^ and STHdh^+/+^ littermate embryos, and and culture conditions, have been described (58). For the described experiment, cells were grown at the permissive temperature for the A58/U19 temperature-sensitive SV40 large-T antigen (33C) at 5% CO_2_. The cells were grown and maintained at 33°C in High-Glucose (25mM) DMEM (Invitrogen, cat# 11995) with 1 mM pyruvate, 10% FBS (Sigma, cat# 12306C), Penicillin-Streptomycin-Glutamine (CellGro, cat# 30-009-CI) and 400 μg/ml of G418 (Gibco, cat# 11811–031). The final concentration of glutamine in the media was 6 mM.

### Mice, genotyping and diet

The B6.*Hdh* ^*Q111/+*^ mice used for this study have a targeted mutation replacing a portion of mouse *Htt* (formerly *Hdh*) exon 1 with the corresponding portion of human *HTT* (formerly *HD*) exon 1, including an expanded CAG tract (originally 109 repeats) and adjacent CCG rich region, as described previously (59). Mice used in the present study were on the C57BL6/J inbred strain background. The targeted *Htt* allele was placed from the CD1 background onto the C57BL6/J genetic background by selective backcrossing for more than 10 generations to the C57BL6/J strain at Jackson laboratories (60). Cohorts of heterozygote and wild-type littermate mice were generated by crossing B6.Hdh^Q111/+^ males with C57BL/6J females. Mice were genotyped and CAG repeat size determined as described in (61). The size of the *Htt* CAG tract ranged from 126 to 135 (mean 131), with no significant differences observed across the groups of this cohort (cohort data summarized in S3 file). Mouse work was conducted in accordance with National Institutes of Health Guide for the Care and Use of Laboratory Animals and was approved by the Massachusetts General Hospital (MGH) Subcommittee of Research Animal Care (SRAC).

Mice were weaned into group cages and assigned randomly to either a high fat (20/20/60% kcal from protein/carbohydrate/fat) or medium fat (20/35/45% kcal from protein/carbohydrate/fat) diet (Research Diets, New Brunswick, NJ). The mice were maintained on this diet from weaning until sacrifice via CO_2_ inhalation followed by decapitation at 236 +/- 7 days. CNS regions and peripheral tissues were dissected and frozen immediately on dry ice. Blood was collected from the heart and plasma isolated by spinning heparanized blood at 5,800 RPM for 10 minutes at 4C. Tissues and plasma was stored at -80C. For LC-MS analysis, tissues were weighed and lysed in precisely 4 volumes of dH_2_O in 2mL round-bottom tubes with a 5mm stainless steel bead by processing 2 minutes at 30hz in the TissueLyser II system (Qiagen).

### LC-MS

Several LC-MS methods were used to obtain metabolite profiles. Lipids were profiled using a 4000 QTRAP triple quadrupole mass spectrometer (AB SCIEX, Framingham, MA) coupled to a 1200 series pump (Agilent Technologies, Santa Clara, CA) and an HTS PAL autosampler (Leap Technologies, Carrboro, NC). Lipids were extracted from 10 μl of plasma, tissue homogenates, and growth media using 190 μl of isopropanol containing 1-dodecanoyl-2-tridecanoyl-sn-glycero-3-phosphocholine as an internal standard (Avanti Polar Lipids, Alabaster, AL). Cell extracts were prepared from 10 cm plates using 4 ml of cold isopropanol (4°C). The samples were centrifuged for 10 minutes (9,000 x g) and the supernatants were transferred to LC-MS autosampler vials. 10 μl of lipid extract were directly analyzed by reverse-phase chromatography using a 150 x 3.0 mm Prosphere HP C4 column (Grace, Columbia, MD) that was eluted isocratically with 80% mobile-phase A (95:5:0.1 vol/vol/vol 10 mM ammonium acetate/methanol/acetic acid) for 2 minutes followed by a linear gradient to 80% mobile-phase B (99.9:0.1 vol/vol methanol/acetic acid) over 1 minute, a linear gradient to 100% mobile phase B over 12 minutes, then 10 minutes of isocratic 100% mobile phase B. Positive ion MS analyses were carried out using electrospray ionization and Q1 scans. The Ion spray voltage was 5.0 kV and source temperature was 400°C.

For analyses of polar metabolites in the positive ion mode, 10 μl of plasma, tissue homogenates, or growth media were extracted using 90 μl of 74.9%/24.9%/0.2% acetonitrile/methanol/formic acid containing the internal standard valine-d8 (Sigma-Aldrich; St Louis, MO). Cell extracts were prepared from 10 cm plates by adding 5 ml of 80% cold methanol (-80°C). Tissue and fluid extracts were analyzed directly while cell extracts (100 μl) were dried using a nitrogen evaporator and resuspended in 100 μl of 10%/67.4%/22.4%/0.18% water/acetonitrile/methanol/formic acid. Samples were centrifuged (10 minutes, 9,000 x g, 4°C) and the supernatants were analyzed using the LC-MS instrument described above. Metabolites were separated using hydrophilic interaction liquid chromatography (HILIC) with a 150 x 2.1 mm Atlantis HILIC column (Waters; Milford, MA) and a gradient program consisting of: 1 minute of isocratic 5% mobile phase A (10 mM ammonium formate and 0.1% formic acid), a linear gradient to 40% mobile phase B (acetonitrile with 0.1% formic acid) over 10 minutes. Positive ion mode MS analyses were carried out using electrospray ionization and multiple reaction monitoring (MRM) scans. MRM parameters (declustering potentials and collision energies) were optimized for each metabolite by infusion of reference standards before sample analyses. The ion spray voltage was 4.5 kV and the source temperature was 425°C.

Negative ion mode analyses of cell extracts and growth media were achieved using an ion paring chromatography method operated on an Agilent 1200/4000 QTRAP MS system. 100 μL of the 80% methanol extract was dried under nitrogen and resuspended in 80 μl of water containing the internal standard phenylalanine-d8 (Cambridge Isotope Labs, Andover, MA). Polar metabolites were separated using a 150 x 2.1 mm Atlantis T3 column (Waters, Milford, MA) with the following elution conditions: isocratic elution with 100% mobile phase A (10 mM tributylamine and 15mM acetic acid) for 4 minutes followed by a linear gradient to 98% mobile phase B (methanol) over 35 minutes. Negative ion mode MS analyses were conducted using MRM scans with a spray voltage at of -4.5 kV and a source temperature of 550°C. Negative ion mode analyses of polar metabolites in tissue homogenates and plasma were conducted using a HILIC chromatography method operated on an ACQUITY UPLC (Waters; Milford, MA) coupled to a 5500 QTRAP (AB SCIEX, Framingham, MA). Samples (30 μl) were extracted with the addition of four volumes of 80% methanol containing inosine-^15^N4, thymine-d4 and glycocholate-d4 internal standards (Cambridge Isotope Laboratories; Andover, MA). The samples were centrifuged (10 min, 9,000 x g, 4°C), and the supernatants are injected directly onto a 150 x 2.0 mm Luna NH2 column (Phenomenex; Torrance, CA). The column was eluted at a flow rate of 400μL/min with initial conditions of 10% mobile phase A (20 mM ammonium acetate and 20 mM ammonium hydroxide in water) and 90% mobile phase B (10 mM ammonium hydroxide in 75:25 v/v acetonitrile/methanol) followed by a 10 min linear gradient to 100% mobile phase A. Negative ion mode MRM analyses were carried out using an ion spray voltage is -4.5 kV and 500°C source temperature. All raw LC-MS data were processed using MultiQuant software (version 1.2, AB SCIEX, Framingham, MA) for peak integration and manual reviewed of data quality.

### Statistics

Metabolite concentrations derived from LC-MS peak areas were analyzed using the R statistical programming language. Additional packages used for specific analytic approaches includes the linear discriminant function (‘lda()’) from the MASS package (62). Many data were graphed using the ggplot2 package (63) and random forest and variable importance analysis were conducted using the ‘randomForest’ package (64). Raw metabolite concentrations for all cells and tissues analyzed are included as S1 and S2 files.

## Supporting Information

S1 File Tissue Metabolite Concentrations. Peak areas for each metabolite (column) for each tissue/mouse combination (row).
(CSV)

S2 File Tissue Metabolite Concentrations. Peak areas for each metabolite (column) for each cell and media sample.
(CSV)

S3 File Metabolite meta-information. Detailed information for each metabolite, including database links and method information.
(XLS)

S4 File Summary cohort characteristics. Measured CAG size is reported as mean (standard deviation) for each sub-cohort.
(DOCX)
