# Supplementary material for: HdhQ111 Mice Exhibit Tissue Specific Metabolite Profiles that Include Striatal Lipid Accumulation
Source: PLoS One. 2015 Aug 21;10(8):e0134465. doi: 10.1371/journal.pone.0134465 (PMC4546654; doi:10.1371/journal.pone.0134465)
Supplement: S4 File — Measured CAG size is reported as mean (standard deviation) for each sub-cohort. (DOCX) [file pone.0134465.s004.docx]

**Supplementary Table 4 –** Summary cohort characteristics. Measured CAG size is reported as mean (standard deviation) for each sub-cohort.

| Gender | Genotype | CAG Size | N |
| --- | --- | --- | --- |
| M | **Hdh^Q111/+^** | 131 (2.1) | 8 |
| F | **Hdh^Q111/+^** | 129 (2.7) | 6 |
| M | **Hdh^+/+^** | 7 (0) | 6 |
| F | **Hdh^+/+^** | 7 (0) | 10 |
